# A case of venous aneurysm of a splenorenal shunt

**DOI:** 10.1259/bjrcr.20210011

**Published:** 2021-07-08

**Authors:** Hiroki Yonezawa, Atsushi Jogo, Akira Yamamoto, Takehito Nota, Kazuki Murai, Satoyuki Ogawa, Mariko Nakano, Ken Kageyama, Shinichi Hamamoto, Etsuji Sohgawa, Masao Hamuro, Toshio Kaminou, Yukio Miki

**Affiliations:** 1 Department of Diagnostic and Interventional Radiology, Osaka City University Graduate School of Medicine, Abeno-ku, Osaka, Japan; 2 Department of Radiology, National Hospital Organization Osaka Minami Medical Center, Kawachinagano-shi, Osaka, Japan; 3 Department of Radiology, Osaka City General Hospital, Miyakojima-ku, Osaka, Japan; 4 Department of Radiology, Izumiotsu Municipal Hospital, Izumiotsu-shi, Osaka, Japan; 5 Advanced Imaging & Minimally Invasive Therapy Center, Tsukazaki Hospital, Himeji-shi, Hyogo, Japan

## Abstract

A 66-year-old man presented with liver cirrhosis due to non-alcoholic steatohepatitis and hyperammonemia. Contrast-enhanced CT showed a dilated and tortuous splenorenal shunt and a large venous aneurysm in the shunt. The venous aneurysm showed gradual enlargement over 10 years and worsening hyperammonemia, so balloon-occluded retrograde transvenous obliteration was performed. Under balloon occlusion, 5% ethanolamine oleate was injected from a microcatheter into the venous aneurysm, which was subsequently embolized with microcoils. Contrast-enhanced CT after the procedure showed complete thrombosis of the venous aneurysm. 10 months later, the venous aneurysm reduced in size, and hyperammonemia had improved.

## Introduction

Portosystemic shunts (PSSs) are formed under conditions of portal hypertension due to cirrhosis and frequently associated with hepatic encephalopathy (HE).^1^ Chronic recurrent HE (CRHE) due to PSS has recently been treated with balloon-occluded retrograde transvenous obliteration (B-RTO).^2^ In this case, B-RTO was performed for hyperammonemia due to splenorenal shunt with localized aneurysmal change in the splenorenal shunt. Venous aneurysm of the splenorenal shunt (“splenorenal shunt aneurysm”) is rare, and we report herein a case with successful endovascular treatment of a splenorenal shunt aneurysm.

## Case presentation

### Clinical course

**Figure 1.**
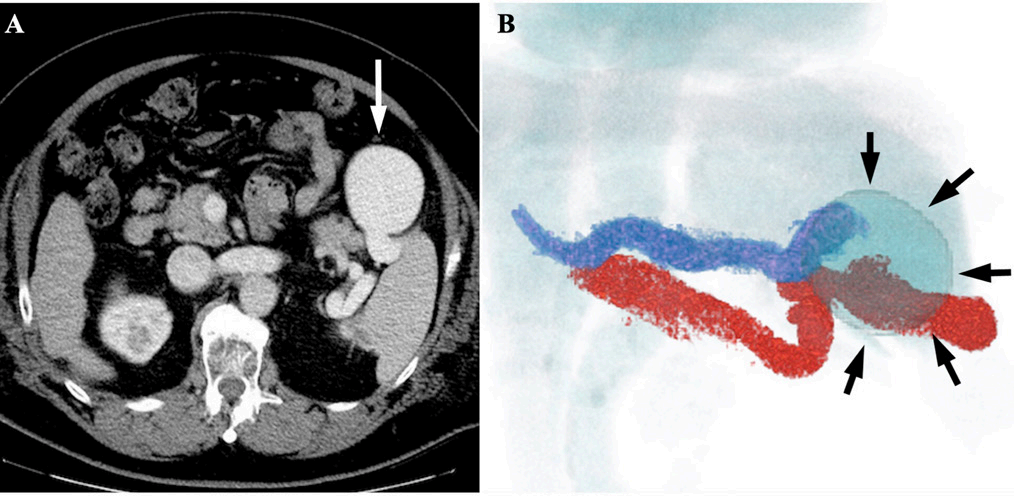
Contrast-enhanced CT image of the portal phase. (**A**) Axial; (**B**) 3DCT. (**A**) Axial image shows a dilated and tortuous splenorenal shunt with a large venous aneurysm (65 mm × 55 mm × 58 mm) (*white arrow*) in the hilus of the spleen. (**B**) On 3DCT, venous aneurysm of the splenorenal shunt (*black arrows*) and feeding and draining veins are clearly recognizable. The feeding vein is the splenic vein (blue line) and the draining vein is the left renal vein (red line).

A 66-year-old male presented with liver cirrhosis due to nonalcoholic steatohepatitis and hyperammonemia. Follow-up contrast-enhanced CT showed a dilated and tortuous splenorenal shunt and large venous aneurysm in the hilus of the spleen (Figure 1A and B). Laboratory data on admission were as follows: erythrocyte count, 488 × 10^4^/mm^3^; hemoglobin, 15.0 g dl^−1^; platelet count, 98 × 10^4^/mm^3^; total bilirubin, 2.9 mg dl^−1^ (elevated); aspartate aminotransferase, 37 IU l^−1^; alanine aminotransferase, 20 IU l^−1^; alkaline phosphatase, 552 IU l^−1^ (elevated); serum ammonia, 125 µg dl^−1^ (elevated); total protein, 6.9 g dl^−1^; serum albumin, 3.6 g dl^−1^; blood urea nitrogen, 11 mg dl^−1^; and creatinine, 0.74 mg dl^−1^. Child-Pugh grade was B (score 7) and albumin–bilirubin (ALBI) grade was 2b (score −1.94). The splenorenal shunt aneurysm had been followed by annual CT for 10 years and gradually enlarged from 20 mm x 27 mm x 24 mm to 65 mm × 55 mm × 58 mm, with an increase of 6 mm in 1 year. Exacerbation of HE was also noted over the previous year. Because the splenorenal shunt aneurysm tended to increase over time and there was a risk of rupture, and HE worsened despite medical therapy, we judged that this was an indication for treatment. The increased splenorenal shunt flow was thought to be one of the causes of the aneurysm enlargement and exacerbation of HE. Therefore, B-RTO was selected to decrease the shunt flow.

## Treatment

### B-RTO

**Figure 2.**
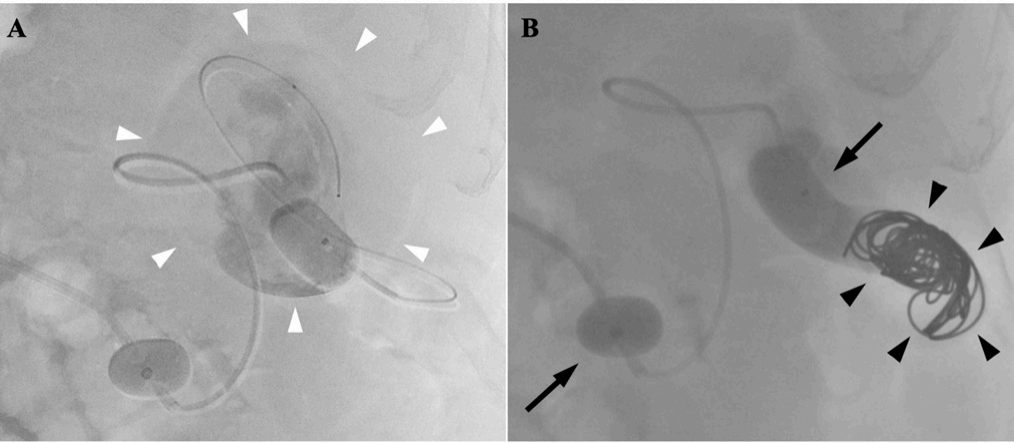
B-RTO (**A**) B-RTV (balloon-occluded retrograde venography) shows venous aneurysm of the splenorenal shunt (*white arrowheads*), laminar flow in the aneurysm and hepatopetal flow in the main portal trunk. A microcatheter is advanced into the aneurysm, and 5% EOI is injected from the microcatheter under balloon occlusion. (**B**) The draining vein is embolized with microcoils (*black arrowheads*) using double-balloon catheter system (*arrows*). B-RTO, balloon-occludedretrograde transvenous obliteration; B-RTV, balloon-occluded retrogradevenography

A coaxial double-balloon catheter system (Candis; Medikit, Tokyo, Japan) was inserted into the splenorenal shunt from the left renal vein via the right femoral vein under local anesthesia. The balloon-occluded retrograde venography showed the portal vein was patent, no thrombosis and the hepatic blood flow was hepatopetal. The microcatheter was advanced into the venous aneurysm (Figure 2A), then 9 ml of 5% ethanolamine oleate (Oldamin; ASKA Pharmaceutical, Tokyo, Japan) was injected from the microcatheter under balloon occlusion. Finally, the draining vein was embolized with microcoils (Figure 2B). We used coils of 1.5 times size in diameter compared to the shunt vein for preventing coil migration. A 5-Fr balloon catheter (9 mm diameter, Selecon MP Catheter II; Terumo, Tokyo, Japan) was inserted into the hepatic vein through the right femoral vein, and pressures were measured using a Polygraph MSC-7000 manometer (Fukuda Denshi, Tokyo, Japan). The measured parameters were right atrial pressure, hepatic venous pressure, and wedged hepatic venous pressure (WHVP). WHVP was 22 mmHg. Under balloon occlusion of the splenorenal shunt, WHVP was 32 mmHg. B-RTO was successfully performed, and no complications were observed.

## Follow-up

**Figure 3.**
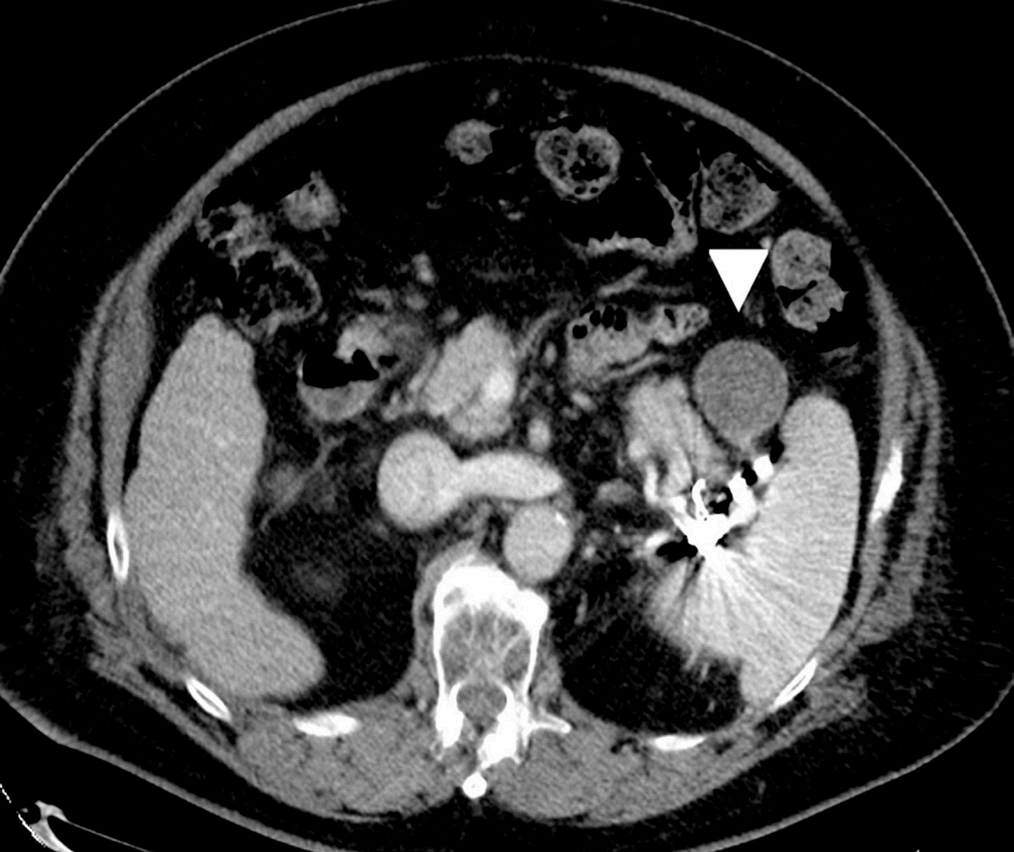
Contrast-enhanced CT image of the portal phase (10 months after B-RTO). Axial image shows the thrombosis and shrinkage of the venous aneurysm (*white arrowhead*). B-RTO, balloon-occludedretrograde transvenous obliteration

10 months later, the venous aneurysm was seen to have shrunk (Figure 3), and hyperammonemia had improved. No esophageal varices or ascites were noted. Child-Pugh grade changed from B (score 7) to A (score 6) and ALBI grade changed from 2b (score −1.94) to 2a (score −2.47).

## Discussion

PSSs are common in patients with portal hypertension due to cirrhosis and develop as portal vein pressure increases.^3^ These shunts can be divided into intra- and extrahepatic shunts, such as gastrorenal shunt, splenorenal shunt, superior mesenteric vein-inferior vena cava shunt, and inferior mesenteric vein-inferior vena cava shunt, and these can also lead to HE.^2^ Splenorenal shunt causes HE due to reflux of venous blood and is the most common cause of HE (60%).^3^ CRHE is often controlled using drugs such as lactulose or rifaximin, but some cases prove refractory to pharmacotherapy. Surgical ligation is reportedly effective for the treatment of CRHE, but B-RTO has been widely adopted in Japan for the management of HE.^4^

No reports have described cases with localized aneurysmal changes in the splenorenal shunt, but several reports have described cases with HE due to large splenorenal shunt. Venous aneurysms included portal system aneurysm (PSA), and splenorenal shunt aneurysm are very similar in terms of portal hypertension.^5–8^ PSA is associated with not only portal hypertension but also an inherent weakness of the vessel wall.^5^ In this case as well, the congenital wall weakness and thinning of the shunt itself were thought to be the main cause of aneurysmal change, with the splenorenal shunt aneurysm subsequently increased by portal hypertension.

Standard treatments for splenorenal shunt aneurysm with HE remain lacking. Careful observation without treatment is often selected for extrahepatic PVA.^6^ Surgical treatments for PSA are often indicated in cases with severe symptoms, thrombus formation, worsening of liver function, and enlargement during follow-up. The rupture of PVA has been reported.^8^ Sfyroeras et al reported the diameter of the ruptured PVA was 2 cm.^9^ Similarly, if splenorenal shunt aneurysm continues to increase, there is a risk of rupture. Splenorenal shunt aneurysm should be treated if symptoms such as HE are present or if the aneurysm tends to be large. The increased splenorenal shunt flow is thought to be one of the causes of the aneurysm enlargement and exacerbation of HE. B-RTO is useful to treat the aneurysm itself and HE with splenorenal shunt closure at the same time. In this case, we treated the patient with B-RTO, resulting in thrombosis of the splenorenal aneurysm and shunt closure. The improvement of HE is mainly due to the effect of shunt closure. Thrombosis and reduction of the splenorenal aneurysm by B-RTO will prevent it from rupturing.

Conversely, increased portal blood flow after shunt embolization can lead to complications such as exacerbation of gastric varicose veins, retention of ascites, and progression of hepatic failure.^10^ The indications for treatment of PSS remain unclear, but pre-operative liver function is one of the most important factors in post-operative complications. This case showed Child-Pugh score 7 (class B), and the increase in WHVP was less than 60% before and after balloon occlusion of the splenorenal shunt. No post-operative complications such as varicose vein exacerbation or retention of ascites were observed.

Some recent reports have described portosystemic shunt syndrome, in which the presence of PSS worsens liver function in the long-term.^3,11^ B-RTO plays a protective role against the lowering of hepatic functional reserve in the long term because portal blood flow increases after B-RTO.^11,12^ In our case, Child-Pugh and ALBI grades changed from Child-Pugh Grade B (score 7) and ALBI Grade 2b (score −1.94) to Child-Pugh grade A (score 6) and ALBI Grade 2a (score −2.47). B-RTO was feasible to improve liver function and to prevent rupture of venous aneurysm.

## Conclusions

B-RTO was feasible as a treatment to improve liver function and prevent rupture of splenorenal shunt aneurysm.

## Learning points

- Portosystemic shunt may show aneurysmal formation / aneurysmal change.
- B-RTO for shunt aneurysm was feasible.
